# Sequential Ag doping of Au_25_^−^ atomically precise nanoclusters induces alternating positive and negative shifts of the HOMO–LUMO gap

**DOI:** 10.1039/d5sc06264k

**Published:** 2025-11-28

**Authors:** Jonathan W. Fagan, Nabiha Syed, Wangshu Wen, Hanna Morales Hernández, Alvaro Muñoz-Castro, Christopher J. Johnson

**Affiliations:** a Department of Chemistry 100 Nicolls Road Stony Brook New York 11794-3400 USA chris.johnson@stonybrook.edu +1 631 632 7577; b Facultad de Ingeniería, Universidad San Sebastián Bellavista 7 Santiago 8420524 Chile alvaro.munozc@uss.cl

## Abstract

Au_25_(SR)_18_^−^  is the most studied atomically precise metal nanocluster and is frequently used to explore the effects of doping on nanocluster performance in a range of applications. A quantitative accounting of the impact of dopants on its electronic structure remains elusive due to the inability to isolate clusters with exact numbers of dopants and the low resolution of experimental techniques used to probe electronic structure. We present high-resolution UV-vis spectra of Au_25−*n*_Ag_*n*_(SC_6_H_13_)_18_^−^ (*n* = 0–6) recorded at low temperature in the gas phase after being precisely separated by mass spectrometry. These spectra feature substructure in the transitions among the frontier orbitals that reveal their shifts upon doping. Ag-doping blue shifts most spectral features, in accordance with theoretical predictions and observations, but the HOMO–LUMO gap shows an alternating pattern of red and blue shifts with increasing Ag substitution. We interpret these spectral shifts using the superatomic (jellium) model as resulting from perturbations to specific superatomic orbitals with nodes or nodal planes oriented with respect to the Ag dopant sites. Quantum chemical results reproduce these observations. These results show an unanticipated quantized doping effect that can be rationalized using accepted intuitive models and serve as clear quantitative tests for ongoing efforts to improve quantum chemical treatments of doped nanoclusters.

## Abstract 2

Unexpected alterneting shifts of frontier orbital energies in the Au_25_(SR)18^−^  nanocluster can be linked to site-dependent perturbations depending on their location with respect to lobes and nodal planes.

## Graphical abstract

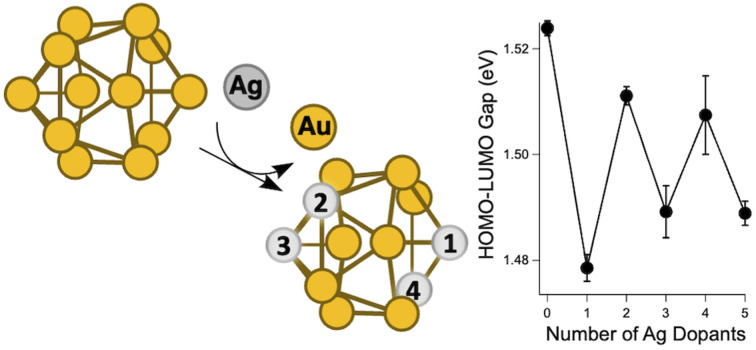

## 1 Introduction

Atomically-precise monolayer-protected clusters (MPCs) have attracted attention as nanoparticles with atomically-precise compositions,^1^ well defined structures,^2^ and potentially high stability.^3^ This precision suggests a path towards the reproducible synthesis of nanoparticles with tailored functionality and broad tunability. These favorable properties have led to their exploration as atomically-tunable light harvesters, catalysts, sensors, and contrast agents, among many potential applications.^4–6^

MPCs consist of a core of metal atoms that is spherical or spheriodal in shape surrounded by organic ligands that stabilize and solubilize the metal core. Typically 1 to 2 nm in diameter, they exhibit a dense but finite molecule-like density of states that often yields distinct features in UV/visible absorption spectra.^7^ The electronic structure of these nanoclusters is typically rationalized using the “superatomic” model, in which electrons are delocalized across the core in orbitals that bear a striking resemblance to hydrogen atomic orbitals.^8^ These orbitals are referred to by their symmetry, following the pattern 1S, 1P, 1D, 2S, 1F, …electronic shell closings similar to those found in atoms or coordination complexes impart particular electronic stability to certain combinations of composition and charge state.^9^ The superatomic model thus provides a qualitative framework for understanding electronic effects in MPCs.

**Fig. 1:**
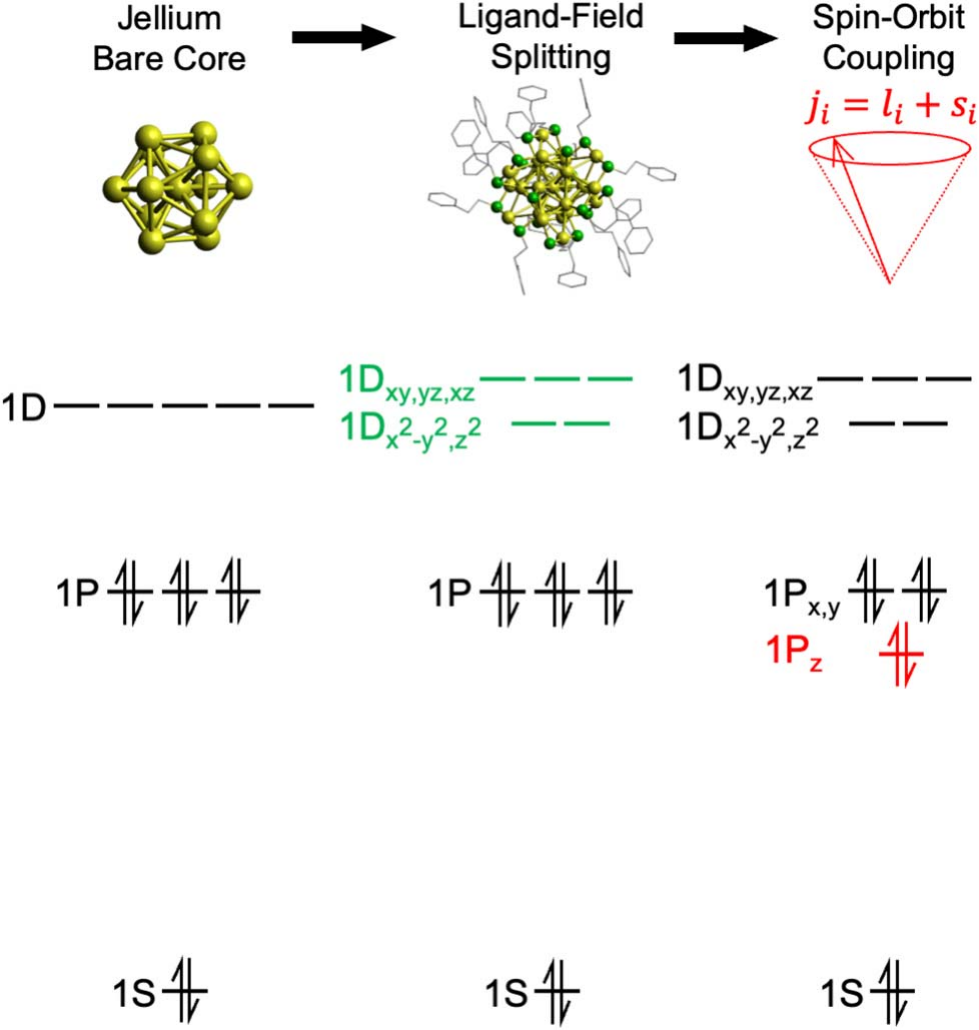
Evolution of the superatom electronic structure of Au_25_(SR)_18_^−^ starting from a simple Au_13_ metal core which can be approximated by the jellium model, followed by splitting of the superatomic D orbitals upon inclusion of the ligand shell, as described by Aikens *et al.*^15^ The final complication of the electronic structure arises from populating the HOMO levels where spin–orbit coupling further breaks the degeneracy of the superatomic P and D orbitals, as depicted in the work of Jiang and co-workers.^16^

Since its confirmation by mass spectrometry^10^ and the subsequent determination of its crystal structure,^11,12^ Au_25_(SR)_18_ has been the most widely studied MPC.^13^ It is composed of an iscosahedral Au_13_ core surrounded by a shell of six Au_2_(SR)_3_ units, the so-called “staple” surface motif. It is commonly found in three oxidation states: −1, 0, and +1.^14^ The electron configuration of Au_25_(SR)_18_^−^, as understood within the superatomic model, is a closed-shell, 8-electron 1S^2^1P^6^ superatom, with unfilled 1D orbitals as low-lying LUMOs,^12^ as shown on the right of Fig. 1.

Despite the fact that the geometric structure of Au_25_(SR)_18_^−^  has been known with high precision for more than a decade, quantifying its electronic structure is more challenging. Au_25_(SR)_18_^−^  has been the subject of numerous spectroscopic and quantum chemical studies; we will specifically highlight a few insights crucial to this work. Initial UV/vis spectra of Au_25_(SR)_18_^−^  showed two main spectroscopic regions: an asymmetric feature below 2.2 eV and a more complex set of features at higher energy.^12^ Aikens used density functional theory (DFT) calculations to demonstrate that, in Au_25_(SH)_18_^−^, the (SH)-Au-(SH)-Au-(SH) staples form a ligand shell around the icosahedral core that breaks the degeneracy of the 1D MOs analogous to ligand-field splitting in octahedrally coordinated monometallic complexes, but with the opposite energy ordering, as shown in the center of Fig. 1.^17^ The result is 1Dx_^2^_−y_^2^_ and 1Dz_^2^_ being lower in energy, and thus the lower energy feature could be readily assigned to transitions from filled 1P orbitals to these low-lying unfilled 1D orbitals. Further insight came from higher-resolution low-temperature spectra, which resolved the asymmetric low-energy feature into at least two distinct transitions.^18^ Jiang and co-workers showed that these two features could be explained by explicit consideration of relativistic effects in DFT calculations, specifically the splitting of the nominally triply-degenerate 1P_*x*,*y*,*z*_ sublevels into two 1P_3/2_ and one 1P_1/2_ orbitals due to spin–orbit coupling, shown to the right in Fig. 1.^16^ Li and co-workers further applied relativistic DFT methods to the model system Au_25_(SH)_18_^−^ and showed that the low-temperature spectrum of Au_25_(SR)_18_^−^  could be satisfactorily explained.^19^ Taken together, these studies lead to high confidence that the low-energy features of the spectrum of Au_25_(SR)_18_^−^  are accounted for, at least semiquantitatively.

Doping^20^ and ligand exchange^21,22^ provide two major pathways to modify the electronic structure of Au_25_(SR)_18_^−^  without inducing major structural changes. Modification of the ligand shell is expected to induce subtle perturbations to the superatomic orbitals due to electrostatic and explicit bonding effects,^15^ which appear to be smaller for thiolate clusters compared to phosphine ones.^23^ Metal doping should produce a stronger perturbations by directly altering the potential of the cluster core, on which the superatomic orbitals primarily reside.^24^ Ag doping of Au_25_(SR)_18_^−^  has been shown to increase photoluminescence quantum yields or^25^ enhance catalytic performance.^26^ Much effort has gone to understanding the electronic perturbations induced by Ag doping of Au_25_(SR)_18_^−^  because Ag dopants typically lead to isovalent clusters with small structural perturbations and a high degree of doping can be achieved.^27–31^ Atomically-precise isolation of clusters with specific numbers of Ag dopants is a significant challenge, impeding efforts to systematically study experimentally the effects of doping one atom at a time. Thus, most experiments probe an ensemble of doped states. Negishi and co-workers used high performance liquid chromatography (HPLC) to partially resolve Au_25−*n*_Ag_*n*_(SR)_18_^−^ nanoclusters and recorded their electronic spectra, finding that the lowest-energy spectral feature progressively blue shifted with increasing doping.^32^ Jin and co-workers found a similar blue shift of the spectrum of a mixture of clusters with 0–5 Ag dopants,^33^ and Stamplecoskie and co-workers found a similar result for photoemission spectra.^34^ Quantum chemical calculations generally find a similar trend, with computed peaks in electronic spectra shifting to higher energy with increasing Ag substitution.^35–38^ They also largely find that the icosahedral vertices are the most energetically favorable sites,^35,38,39^ consistent with analysis of X-ray absorption fine structure data and perturbations to X-ray crystal structures induced by dopants.^28,29^

In this study, we track the evolution of the high-resolution electronic spectra of Ag-doped Au_25_(SR)_18_^−^  nanoclusters one Ag atom at a time. We employed a spectroscopic technique that allows us to use mass spectrometry to arbitrarily purify nanoclusters and then, in the gas phase, record their electronic^40^ and vibrational^41^ spectra at low temperature. This method provides spectroscopic resolution close to the quantum limit, revealing an alternating pattern of red and blue shifts of the HOMO–LUMO gap that has not been previously observed.

## 2 Materials and methods

Au_25_(SR)_18_^−^  and Au_25−*n*_Ag_*n*_(SC_6_H_13_)_18_^−^  were synthesized according to modified literature procedures^12,14,42^ as outlined in the SI. The dopant ratios of the doped clusters were roughly controlled by equilibrating mixtures of the two products as discussed in detail in the SI, following an intra-cluster exchange method reported by Pradeep and co-workers.^43^ Samples were dissolved in acetonitrile or acetone and electrosprayed in a home-built quadrupole/ion trap/time of flight mass spectrometer described previously.^44^ Here, the mass spectrometer acts as a general purification device, straightforwardly isolating any doped cluster in the gas phase as an alternative to challenging condensed phase separations.^32^ While in the mass spectrometer, each doped cluster studied was cooled to 3.6–3.8 K and its unique UV/visible spectrum was recorded using an action spectroscopy scheme discussed previously,^40^ with specific details explained in the SI. Explanations of the data analysis and fitting procedures are also provided in the SI. 

## 3 Results and discussion

**Fig. 2:**
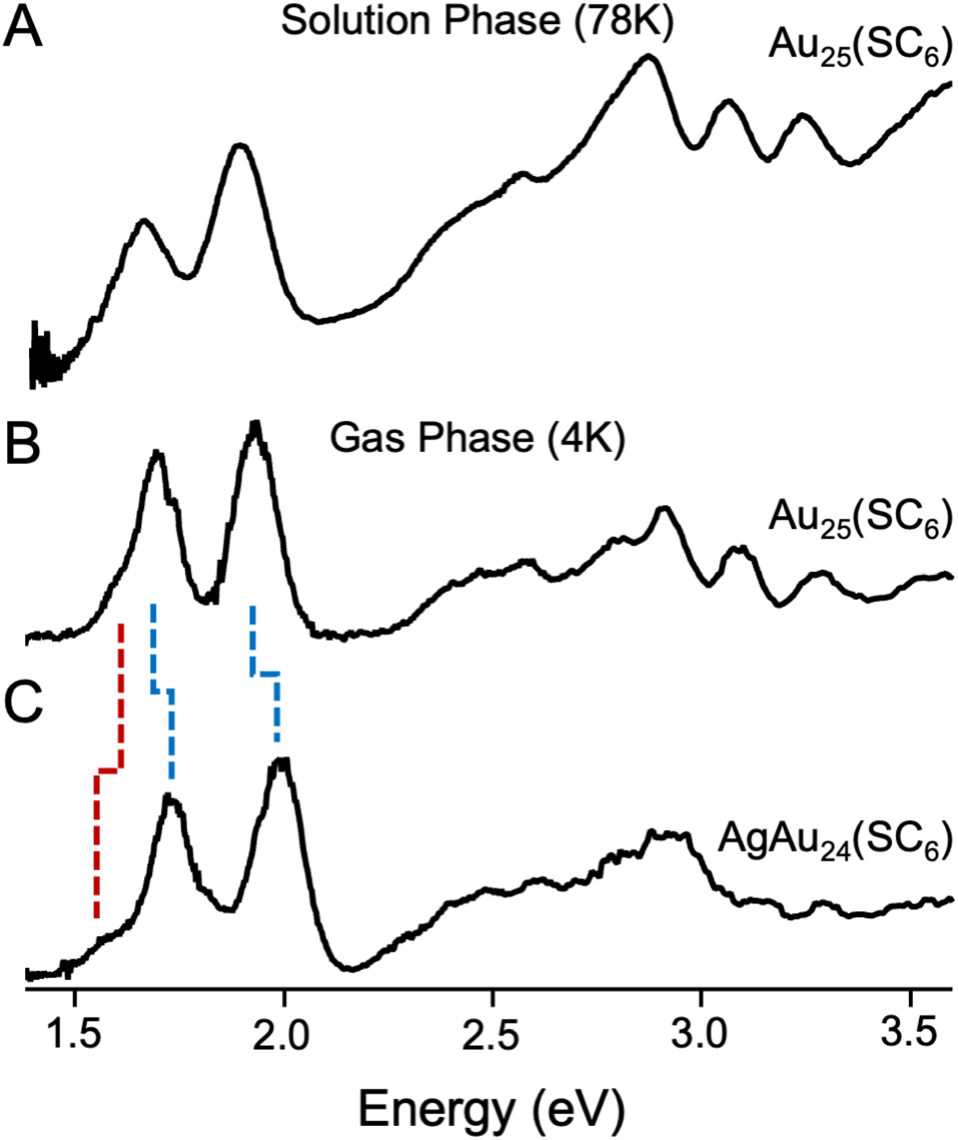
(A) Solution phase UV/vis spectrum of Au_25_(SC_6_H_13_)_18_^−^  collected at 78 K and reported by Devadas *et al.*,^18^ (B) gas phase UV/vis spectrum of Au_25_(SC_6_H_13_)_18_^−^ at 4 K, and (C) gas phase UV/vis spectrum of Au_24_Ag(SC_6_H_13_)_18_^−^  at 4 K. Dashed blue lines denote peaks that blue shift, while dashed red lines denote peaks that red shift. The extra resolution afforded by the lower temperature gas phase spectra reveals substructure of both clusters that we analyze in this work.

Fig. 2 presents an overview of the spectra of Au_25_(SR)_18_^−^  in two different conditions: (A) Au_25_(SC_6_H_13_)_18_^−^  in solution at 78 K^18^ and (B), Au_25_(SC_6_H_13_)_18_^−^  in the gas phase at 3.6 K as reported in this work. The low-temperature solution phase UV-vis spectrum of Au_25_(SC_6_H_13_)_18_^−^ in Fig. 2A was the highest-resolved spectrum of Au_25_ to date in the literature. The new gas phase spectrum of Au_25_(SC_6_H_13_)_18_^−^  in Fig. 2B is consistent with the 78 K spectrum, with additional detail in the lower energy transitions. We particularly highlight substructure in the lowest-energy transition, notably a partially resolved shoulder near 1.6 eV that we identify as the HOMO–LUMO transition and a partially-resolved shoulder near 1.8 eV on the high energy side of the feature. The slight blue shifting of our gas phase spectrum compared to the solution phase one is attributed to the absence of solvation effects. We have attributed the breadth of the transitions in these low-temperature gas phase spectra to the excitation of a broad envelope of unresolvable vibrational excited states in the electronic excited state upon optical absorption, convolved with the expected picosecond-to-femtosecond lifetimes of these excited states.^40^ This suggests that these spectra are resolved to the limit of resolution, though we are unable to rule out inhomogeneous broadening effects induced by slightly different ligand packing structures.

We further recorded gas-phase UV-vis absorption spectra of the analogous AgAu_24_ cluster, shown in Fig. 2C. Both gas phase UV/vis spectra exhibit broadly similar features but notable perturbations. The peaks of the two major features around 1.7 eV and 2.0 eV overall shift to higher energy by 0.03 eV and 0.07 eV, consistent with previous spectroscopic observations,^32^ but the apparent HOMO–LUMO transition visually red shifts upon incorporation of the Ag atom. Above 2.2 eV, the spectra of the two clusters remain fairly consistent, with somewhat less definition in the Ag-doped spectrum. To confirm that these observations were robust, we recorded the spectra of the same clusters protected by phenylethanethiol. As can be seen in Fig. S12, the shifts induced by substitution of ligands are much smaller than those induced by doping.

**Fig. 3:**
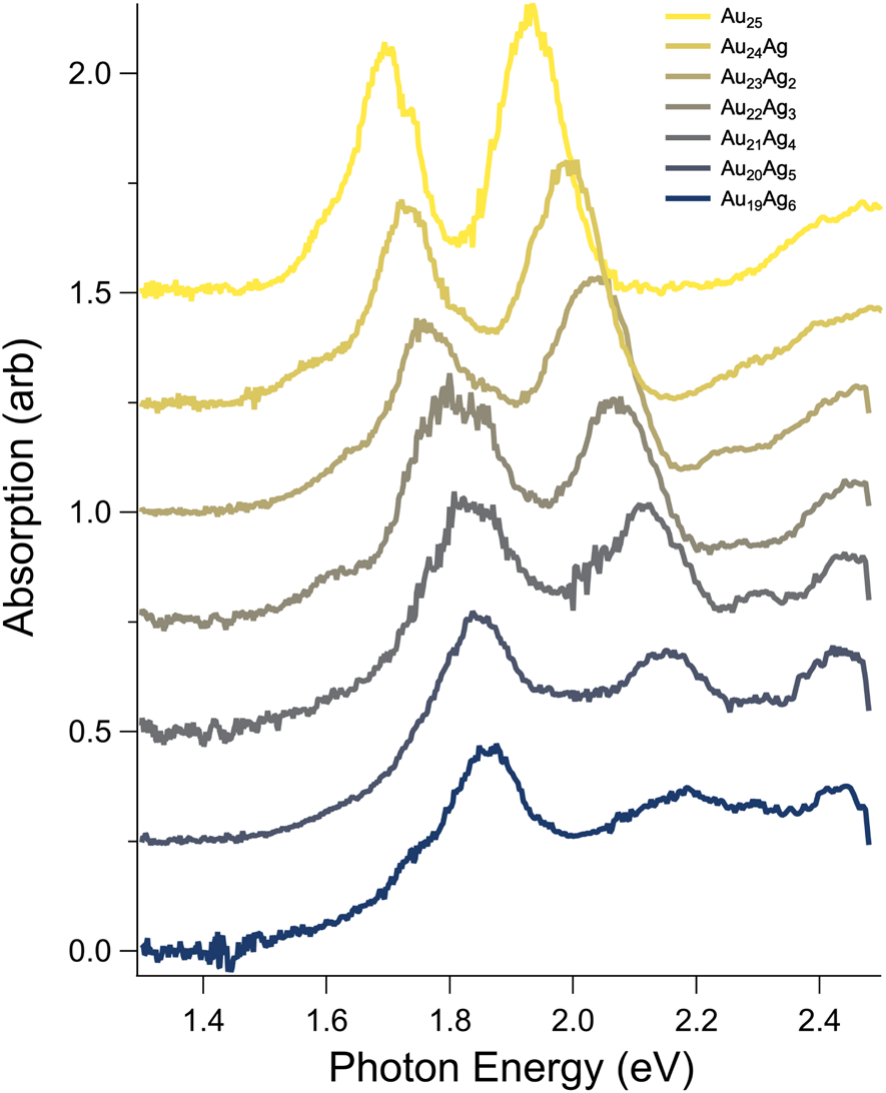
Evolution of the spectra of Au_25−*n*_Ag_*n*_(SC_6_H_13_)_18_^−^  (*n* = 0–6) atomically-precisely separated by mass spectrometry. Most spectral features blue shift with increasing Ag content, but the apparent HOMO–LUMO transition shows more complex behavior.

Fig. 3 presents the spectra of mass-spectrometrically-purified Au_25−*n*_Ag_*n*_(SC_6_H_13_)_18_^−^  up to *n* = 6, focusing on the low-energy region of the spectrum typically assigned to transitions among frontier orbitals. The first four dopants yield approximately linear shifts of the two main peaks in the spectra, matching previous results.^32^ From *n* = 4 to *n* = 6, only very small shifts of the lower energy peak are observed, while the higher-energy peak continues to blue shift at roughly the same rate. Interestingly, the intensity of the higher energy peak is modulated by Ag doping, initially slightly increasing in intensity up to *n* = 2, then rapidly decreasing to *n* = 6. The region above 2.1 eV evolves from a broad, featureless shoulder beginning around 2.2 eV into a pair of peaks that appear to shift similarly to the lower-energy features with increasing doping but significantly increase in intensity. Given that their intensity appears to be proportional to the number of dopant atoms, we tentatively assign these peaks to transitions arising from Ag d-orbitals to low-lying unoccupied superatomic orbitals.

**Fig. 4:**
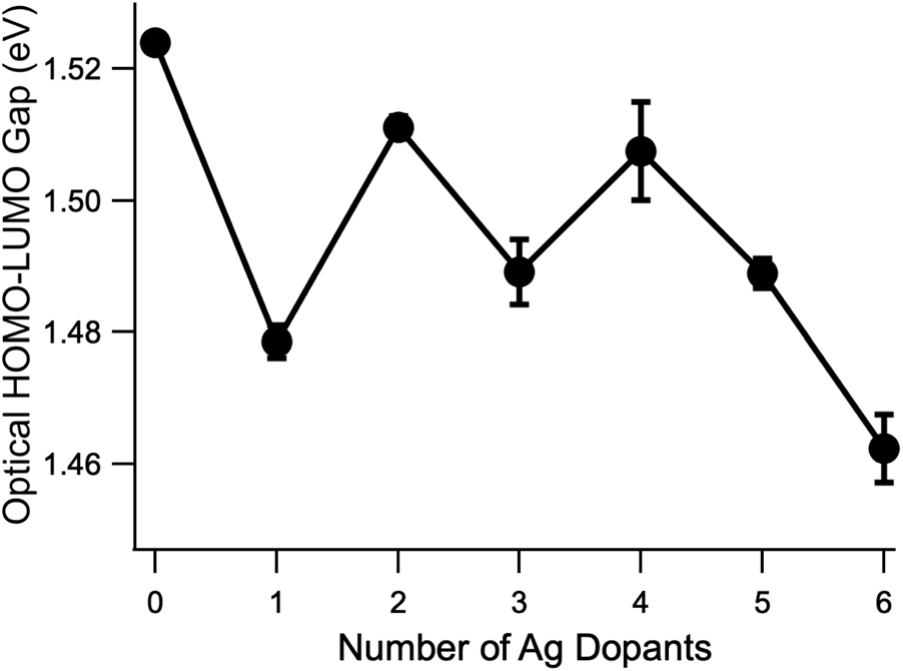
Evolution of the extrapolated optical HOMO–LUMO gap as a function of the number of dopant Ag atoms. Error bars as shown represent the uncertainty of the fit performed to determine the *x*-intercept of the linear extrapolation. Details of the procedure to extract these values are given in the SI. An odd-even red/blueshifting pattern is observed for the first five doped clusters.

Focusing on the shoulder of the lowest-energy peak identified as the HOMO–LUMO transition, which red shifted from Au_25_(SC_6_H_13_)_18_^−^  to Au_24_Ag(SC_6_H_13_)_18_^−^, we observed a pattern of alternating red and blue shifts that appear to correlate to odd and even numbers of Ag atoms, respectively. To quantify these shifts, we estimated the optical HOMO–LUMO gap by extrapolating the linear portion of the shoulder to zero absorption using a linear fit. The results of these fits are presented in the SI and the extrapolated optical HOMO–LUMO gaps are summarized in Fig. 4. The alternating red and blue shifts are clearly resolved, with shifts of 0.02 to more than 0.04 eV observed. Notably, these shifts are greater than *kT* at room temperature (0.025 eV), suggesting that they should still be important in typical solution conditions. This pattern appears to extend to *n* = 5, but the HOMO–LUMO gap of Au_19_Ag_6_ continues to decrease, suggesting a more complex situation for six dopants.

We sought to confirm this even-odd pattern and to track the evolution of the other features in these spectra by decomposing them into individual Gaussian contributions. Neglecting relativistic effects, we expect six transitions to contribute to these peaks arising from transitions from three filled orbitals to two unfilled orbitals. Thus, we fit the spectra in the range from 1.3 to 2.3 eV to a model consisting of the sum of six Gaussian functions for the two dominant peaks, a constant background, and an additional Gaussian function to approximate the putative transitions arising from Ag d-orbitals. The details of the fitting procedure and the results of these fits are presented in the SI. Satisfactory fits were achieved for *n* = 1–5, with the positions, widths, and amplitudes of each Gaussian component being conserved or evolving smoothly from one doped cluster to the next. We were not able to achieve a reliable fit for Au_19_Ag_6_(SC_6_H_13_)^−^ in the same way, likely due to interference from the putative Ag d-orbital based transitions, and we omit it from our analysis.

**Fig. 5:**
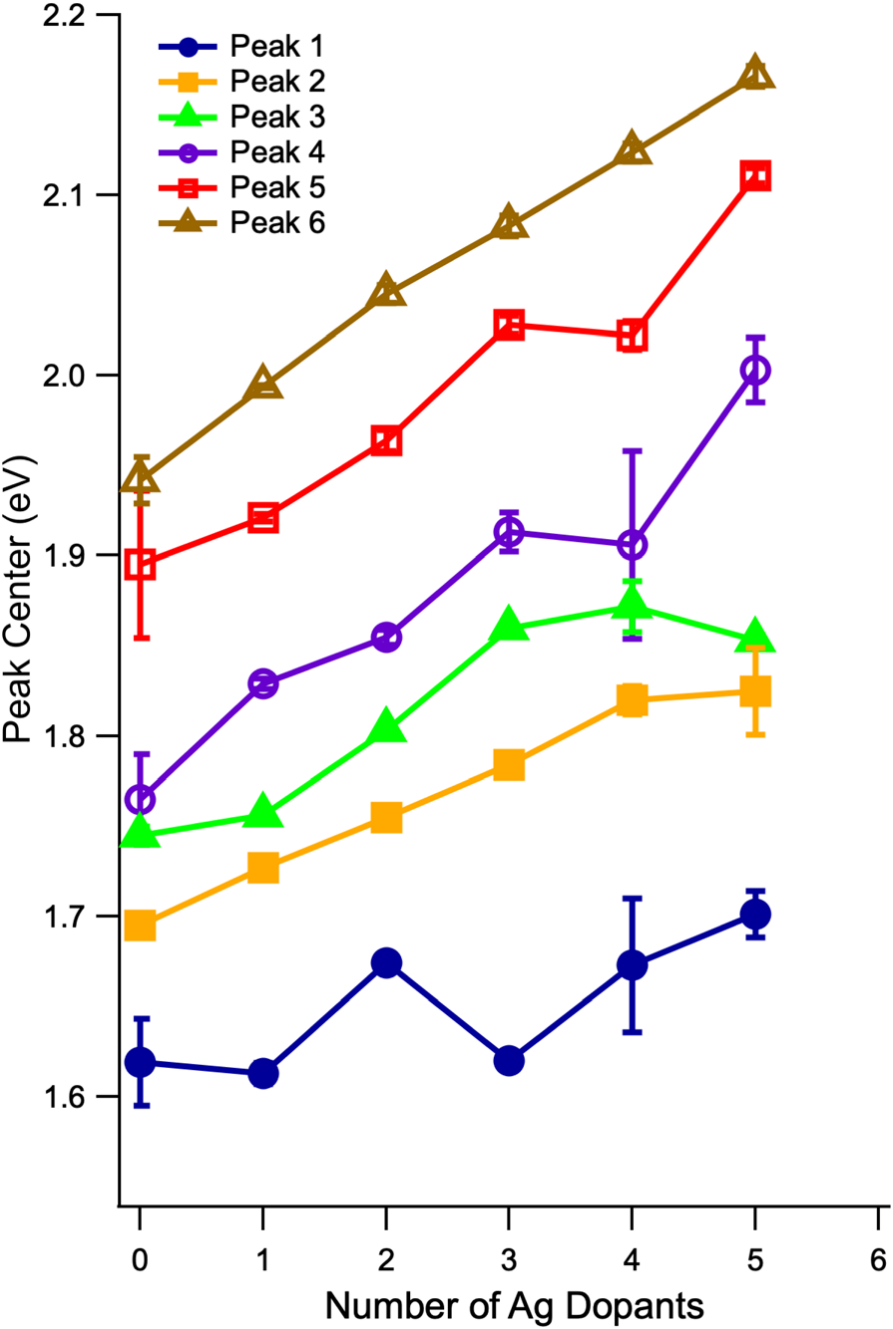
Evolution of the centers of the partially-resolved transitions in the low-energy region of the spectrum, extracted by fitting each spectrum to six Gaussian functions. The center of the presumed HOMO–LUMO transition follows the alternating pattern of red and blue shifts observed for the optical HOMO–LUMO gap in Fig. 4. A reliable fit could not be obtained for the case of 6 Ag dopants.

The evolution of the centers of each Gaussian component as a function of the number of Ag atoms is shown in Fig. 5. First, we see that the odd/even alternating red and blue shifts of the HOMO–LUMO transition from Fig. 4 are also observed in the band centers of the first peak, corresponding to the vertical HOMO–LUMO transition energies. The best fitting widths for the first peak vary between 0.1 and 0.25 eV (full-width at half max). We typically interpret these widths as reporting on the difference between the ground and excited state minimum energy geometries, and this wide range of widths suggests that the S_1_ geometries are quite different from one dopant number to the next. Aikens and co-workers predicted a significant change in the S_1_ geometries of Au_25_(SH)_18_^−^ and Au_24_Ag(SH)_18_^−^.^37^ Second, we note essentially monotonic blue shifts for the other five transitions, in accordance with previous studies.^27^ The fitted peak widths for these features vary much less than do those of the HOMO–LUMO transition, and thus it does not appear that there is as much variation in the more highly excited state geometries. Quantum chemical calculations suggest that, even for Au_23_Ag_2_(SH)_18_^−^, the energies of isomers containing Ag atoms at different relative locations are close enough that it could be possible for multiple isomers to exist in solution at room temperature.^35,38^ The fact that we are able to decompose each of the spectra up to *n* = 5 using six Gaussian functions, each having similar widths and smoothly evolving relative amplitudes, as well as the qualitative similarity of the spectra with increasing doping, suggests (but by no means proves) that the spectra that we present here result predominantly from a single isomer. Future modifications to the instrument used for these studies should make possible the recording of isomer-selective electronic spectra in a similar way to the existing analogous method for vibrational spectra.^45^

These spectral shifts allow us to speculate on the sites at which doping occurs. Starting with Au_24_Ag^−^, three possible isomers exist: Ag in the outer 12 atoms of the superatomic icosahedron, Ag in the center of the icosahedron, or Ag in the ligand “staple” motifs. Careful examination of computed absorption spectra from Aikens and co-workers^35,39^ and Munoz-Castro and co-workers^38^ shows that both have predicted red shifts upon doping with a single Ag atom that have magnitudes similar to that seen here, though the exact values that they predict were not reported and the red shifts were not explicitly addressed. Some other studies have computed blue shifts for the HOMO–LUMO gap,^37^ indicating that there is not yet a consensus on this expectation.

Tsukuda and co-workers have described an approach to using intuition from the particle in a box model to understand perturbations to cluster electronic structure induced by dopants.^24^ The cluster core is modeled as a spherical square well and the dopant atom as a local dip or rise in the potential at the bottom of the well, related to its ionization potential relative to that of gold. We showed that explicit solution of such a framework through numerical solutions of the particle in a box for an asymmetric ellipsoidal potential were able to capture the energies of absorption features of Au_8_ and Au_9_ clusters with reasonable accuracy.^46^

In this framework, if the Ag atom was at the center, we would expect all 1P orbitals to be similarly perturbed because each orbital's electron probability density overlaps similarly with the dopant location, and thus consistent shifts to all bands would be observed. If the Ag atom was located in a staple, the higher energy transitions above 2.4 eV should be considerably altered without significantly changing lower energy superatomic-based transitions because their orbitals do not substantially explore the ligand shell. If the Ag atom was located at the surface of the icosahedron, the asymmetric modification of the potential in the Jellium superatomic model would produce the greatest variations in the spectra, as one or two of the 1P orbitals would be expected to be significantly more perturbed.

**Fig. 6:**
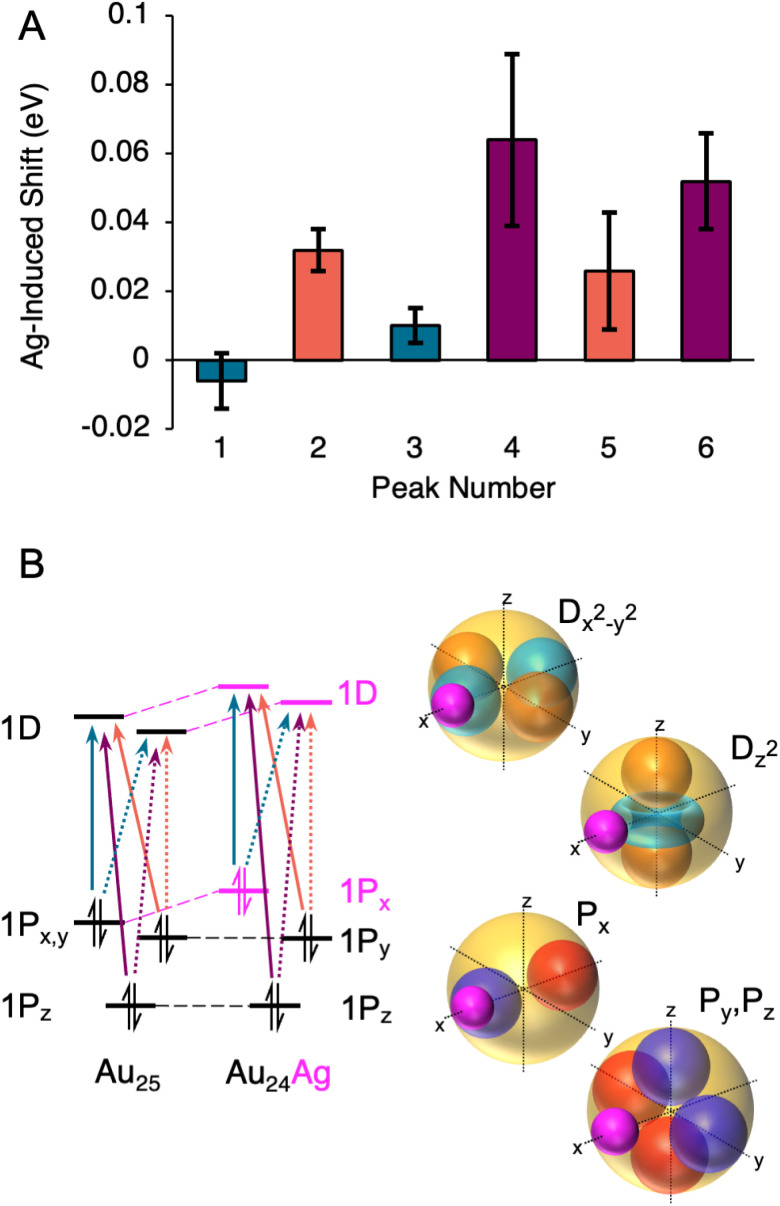
(A) Shifts of the six Gaussian components from Au_25_(SC_6_H_13_)_18_^−^  to Au_24_Ag(SC_6_H_13_)_18_^−^. Bars are colored in three groups according to the magnitude of the shift, which we hypothesize to indicate which of the three 1P superatomic orbitals the transition originates from. (B) A hypothetical transition diagram and pictoral representations of the geometric overlap of the Ag doping site (pink) and the superatomic orbitals.

Fig. 6A shows the shifts induced by the first Ag dopant for each fitted Gaussian component. Three pairs of transitions are noted: one that has small red shifts, one that has moderate blue shifts, and one that has large blue shifts. The observation of three groups of two transitions identifies perturbations to the 1P orbitals as the drivers of the alternating shifts: each 1P sub-level has transitions to two unfilled 1D sub-levels. If the 1P sub-levels were changing in energy similarly, but the 1D sub-levels were shifting differently upon doping, we would instead expect two groups of three transitions. Thus, we conclude that the 1D sub-levels rise approximately equally in energy upon the first dopant, the HOMO (which we assign to the 1P_*x*_ sub-level) rises in energy even more, and the 1P_*y*_ and 1P_*z*_ sub-levels remain roughly the same. This scenario is shown diagramatically in Fig. 6B.

This observation can be explained by the dopant atom lying along a lobe of the 1P_*x*_ orbital, as shown in Fig. 6. The ionization potential of Ag is 1.65 eV lower than that of Au, and thus the Ag atom will appear (within the particle in a box model) as a potential energy bump in the superatomic spherical square well,^24^ and will raise the energies of any orbitals with electron probability density on the Ag atom. Since the 1P_*y*_ and 1P_*z*_ orbitals are in principle orthogonal to 1P_*x*_, the Ag atom will lie along their nodal plane, and only a small perturbation would be expected. The 1Dx_^2^_−y_^2^_ and 1Dz_^2^_ orbitals both have lobes along the Ag dopant, and thus both are shifted to higher energy. Thus, we assign transitions 1 and 3 to transitions from 1P_*x*_ to the 1D sublevels, and transitions 2, 4, 5, and 6 to transitions from 1P_*y*_ and 1P_*z*_ to the 1D sublevels. This explains the red shift of the HOMO–LUMO in conjunction with the blue shift of the 1P → 1D transitions. Such an alignment of a single 1P orbital along the axis of doping was predicted for Au_19_Ag_6_− clusters, in which the dopants were found to occupy opposite sides of the cluster in two groups of three.^28^

**Fig. 7:**
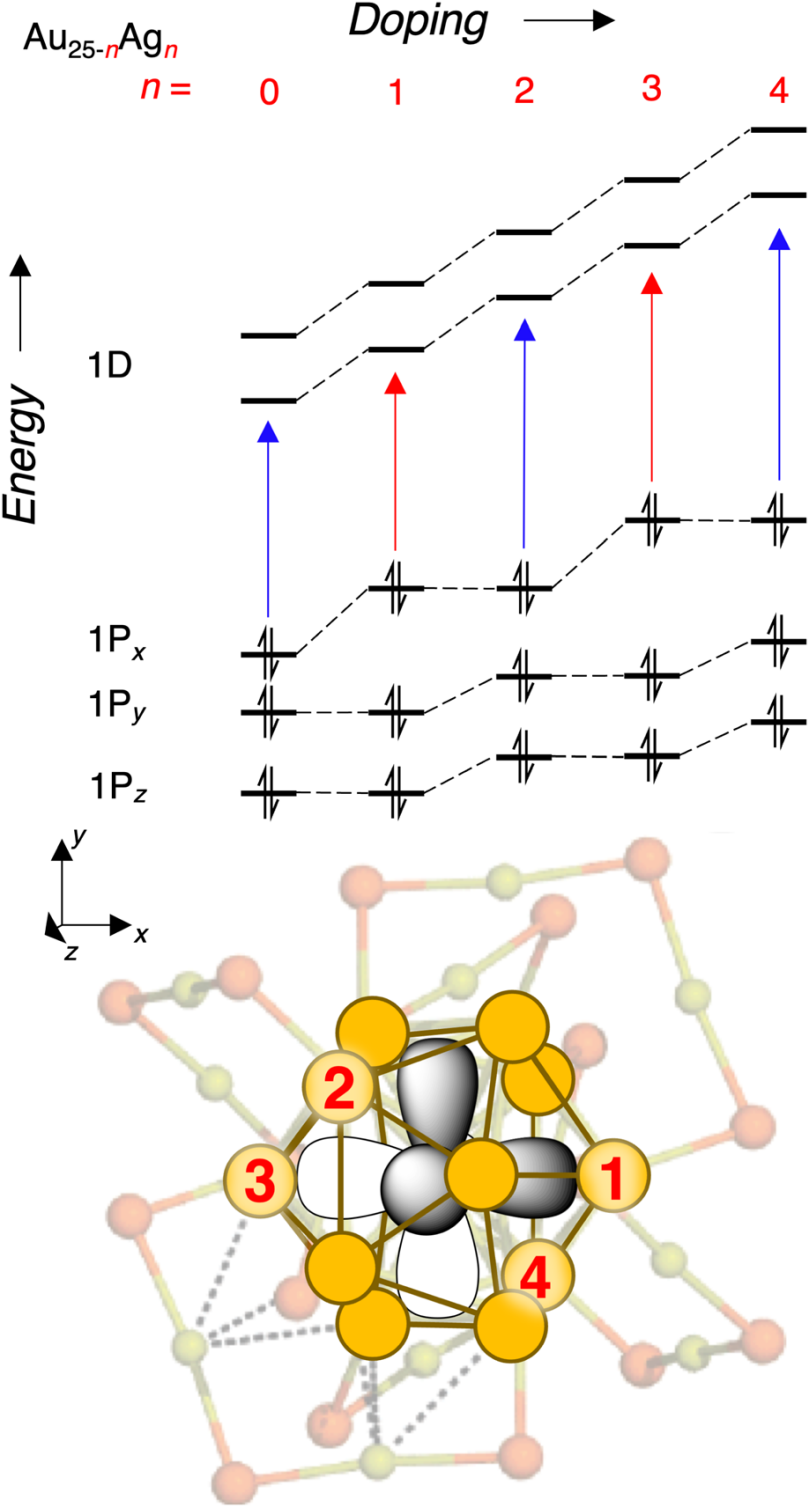
A qualitative model to explain the even-odd pattern of shifts of the HOMO–LUMO gap. Here, odd numbers of dopants are presumed to raise the energy of the 1P_*x*_ superatomic orbital, while even numbers of dopants raise the energies of 1P_*y*_ and 1P_*z*_ orbitals. Since their effect is spread across two orbitals, each even number dopant shifts the *y* and *z* orbitals by less than the odd number dopant shifts the *x* orbital. This model also accounts for the overall increase of the main peaks in the spectrum, with the 1D orbitals shifted by roughly the same amount for both even and odd dopants. At the bottom is a graphical representation of the hypothesized order of doping in the core.

We finally seek an explanation for the alternating red and blue shifts with increasing doping. Following the logic for the first dopant, we propose that the second dopant perturbs the 1P_*y*_ and 1P_*z*_ orbitals roughly equally while leaving 1P_*x*_ relatively unchanged. Again, the 1D sub-levels rise in energy regardless of the location of the second Ag dopant, thus giving rise to a larger blue shift of the transitions originating from 1P_*x*_ and a smaller blue shift of the rest of the transitions. Then, we propose that the third dopant again lies along the 1P_*x*_ orbital, leading to a similar situation as the first dopant, and this pattern seems to continue up to the fifth dopant according to Fig. 4 and 5. This explanation is summarized diagrammatically in Fig. 7. Also in Fig. 7 is a graphical representation on the structure of the Au_25_ core with specific sites labeled in the order that we hypothesize that they are substituted by Ag atoms. This proposed doping order is not intended to be an absolute assignment, but rather to illustrate the explanation we have put forward for alternating perturbations to the P_*x*_ and P_*y*,*z*_ orbitals respectively. The proposed order is broadly consistent with that proposed by Knoppe and Munoz-Castro,^38^ though their calculations suggest that the minimum-energy structure of Au_21_Ag_4_ can not be reached by substitution of one additional Ag atom into the minimum-energy structure of Au_22_Ag_3_.

**Fig. 8:**
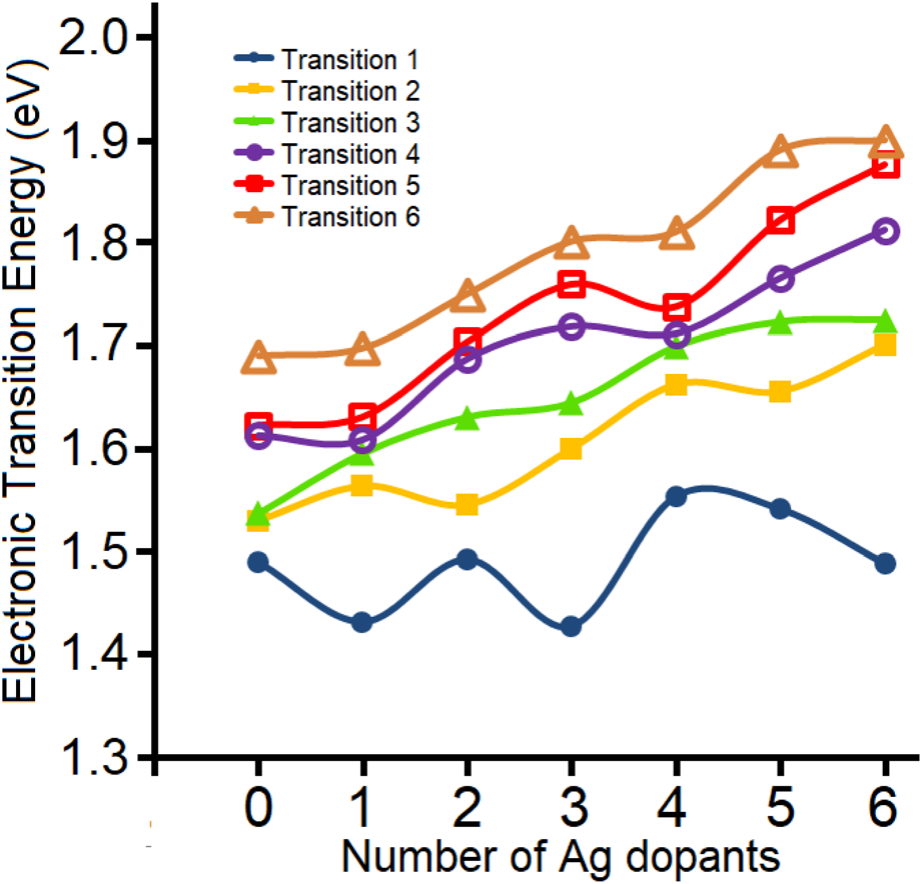
Calculated electronic transitions for the first six transitions of the Au_25−*n*_Ag_*n*_(SH)_18_^−^ clusters. See the SI for details on these calculations.

To further support the experimental findings from the gas-phase UV-vis absorption spectra, TDDFT calculations were carried out on the most energetically-preferred isomers for different stoichiometries of Au_25−*n*_Ag_*n*_(SH)^−^ as determined previously.^38^ The calculated first electronic transition accounting for the lower HOMO → LUMO excitation, shows a variation from Au_25_ to Au_19_Ag_6_ given by 1.49, 1.43, 1.49, 1.43, 1.55, 1.54, and 1.49 eV, respectively (Fig. 8), recovering the even-odd pattern observed experimentally. The calculated values lie within the typical errors of TDDFT calculations relative to experiments.^47^ In addition, up to *n* = 5, the experimental findings are well reproduced by using the energetically-preferred isomer for Au_25−*n*_Ag_*n*_ clusters (*n* < 6), suggesting that these characterized gas-phase species are present as single isomers.

For Au_19_Ag_6_ (*n* = 6), several isomers fall within 1 kcal mol^−1^, suggesting the possibility of a mixture of isomers in the experimental gas-phase characterization. This may explain the difficulty in decomposing the low-lying absorption-spectrum peaks into several Gaussian contributions. For the previously denoted most favorable Au_19_Ag_6_ isomer^38^ (Fig. S13), a lower HOMO → LUMO electronic transition is calculated at 1.63 eV, which does not agree well to the observed optical HOMO–LUMO gap trend, where the isomer at 0.75 kcal mol^−1^ above in energy is able to reproduce the observed gap trend with a calculated value of 1.49 eV, which is discussed in this work to provide TDDFT calculations results. Thus, the current gas phase UV-vis absorption provides a sensitive tool able to discriminate between different low-lying isomers.

The calculated second electronic transition shows a less marked even-odd variation, with a trend toward increasing the gap between the orbitals involved in the transitions as Ag doping increases. Similarly, the third and remaining electronic transitions show a trend toward increasing energy, suggesting that, for such species, the first transition is more sensitive to dopant variation and thus a useful probe for discriminating between different stoichiometries and related isomers.

## 4 Conclusion

We have reported the high resolution gas phase spectra of atomically-precisely separated Au_25−*n*_Ag_*n*_(SC_6_H_13_)_18_^−^  nanoclusters revealing the changes to their electronic spectra induced by doping. While the largest peaks in the spectra blue shift, in accordance with previous theoretical and experimental results, we find that the apparent HOMO–LUMO gap actually shows an alternating pattern of red and blue shifts up to at least *n* = 5. These alternating shifts are large enough to play a role in room temperature chemistry involving these clusters. We observe new bands appearing slightly higher in energy than the transitions among the frontier orbitals of the cluster, and note that the increasing intensity of these bands with increasing doping suggests that they originate from Ag d-orbitals. The resolution of the intra-frontier-orbital region of the spectra provides enough detail to make assignments of the underlying transitions that are consistent with the predictions of six transitions between three superatomimc 1P orbitals and two superatomic 1D orbitals, all of which are non-degenerate. We proposed an interpretation of these observations in which dopant atoms lying along the lobes of superatomic MOs lead to increases in those orbitals energies and thus red shifts in the transitions originating from those orbitals. This interpretation is sufficient to explain the observed alternating red and blue shifting of the HOMO–LUMO gap. Quantum chemical predictions of the electronic transition energies for the lowest-energy computed isomers reproduce the observed pattern well. A similar approach used with other dopants, particularly copper, could yield further insight into the degree to which the electronic properties of these clusters can be intuitively engineered by doping.

## Author contributions

JWF and NS conceived of experiments, recorded spectra, analyzed data, and wrote the manuscript. WW and HMH recorded and analyzed data. CJJ conceived of experiments, analyzed data, and wrote the manuscript. AMC performed quantum chemical calculations and wrote the manuscript.

## Conflicts of interest

There are no conflicts to declare.

## Supplementary Material

SC-017-D5SC06264K-s001

SC-017-D5SC06264K-s002

## Data Availability

The spectral data supporting this article have been included as part of the supplementary information (SI). Supplementary information: including synthetic and experimental details, data analysis details, mass spectrometry results, HOMO–LUMO gap extrapolation results, spectral decomposition results, comparison of spectra with hexanethiol ligands and phenylethanethiol ligands, and computational results for two isomers of Au_19_Ag_6_. See DOI: https://doi.org/10.1039/d5sc06264k.
