# Supplementary material for: Sequential Ag doping of Au25− atomically precise nanoclusters induces alternating positive and negative shifts of the HOMO–LUMO gap
Source: Chem Sci. 2025 Nov 28;17(4):2348–55. doi: 10.1039/d5sc06264k (PMC12683540; doi:10.1039/d5sc06264k)
Supplement: SC-017-D5SC06264K-s002 [file SC-017-D5SC06264K-s002.pdf]

Supplementary Information (SI) for Chemical Science.
This journal is © The Royal Society of Chemistry 2025

Sequential Ag doping of Au_{25}^- atomically precise nanoclusters induces alternating positive and negative shifts of the HOMO-LUMO gap

Jonathan W. Fagan,[†,¶] Nabiha Syed,[†,¶] Wangshu Wen,[†] Hanna Morales Hernández,[†] Alvaro Muñoz-Castro,[‡] and Christopher J. Johnson[*,†]

†*Department of Chemistry, Stony Brook University, Stony Brook, NY*

‡*Facultad de Ingeniería, Universidad San Sebastián, Bellavista 7, Santiago, 8420524,Chile.*

¶*These authors contributed equally to this work.*

E-mail: chris.johnson@stonybrook.edu

Synthetic Details

Syntheses of $\mathbf{MAu_{24}(SC_6H_{13})_{18}^-}$ clusters

All nanoclusters were synthesized by a modified approach based on published protocols.[1,2]

$\mathbf{Au_{25}(SC_6H_{13})_{18}^-}$

$HAuCl_4{\cdot}3H_2O$ (0.51 mmol, 200 mg) was dissolved in THF (20 mL) with tetra-octylammonium bromide (0.61 mmol, 333.21 mg), and the solution was rapidly stirred for 10 minutes. When the solution was deep red, the reaction flask was chilled in an ice bath. Once the reaction mixture reached 0°C the thiol of choice (3.06 mmol) was added drop-wise under slow stirring (c. 50 rpm). The reaction was left to stir slowly over an ice bath for 1 hour, at which time

the reaction had lost all color. After the color loss, the spinning speed was increased (c. 1000 rpm) and a freshly prepared, chilled solution of $NaBH_4$ (5.08 mmol, 192.11 mg) in ethanol (4 mL) was added all at once. The reaction mixture turned black immediately upon the addition of $NaBH_4$ and was left to stir overnight in the ice bath. Large particles and precipitates were removed by centrifugation (3000 rpm for 5 minutes), and THF was removed by rotary evaporation. The residual solid was washed repeatedly with methanol until the scent of unreacted thiol abated. $Au_{25}(SR)_{18}$ clusters were extracted with acetonitrile and confirmed by UV-vis spectroscopy and ESI-MS.

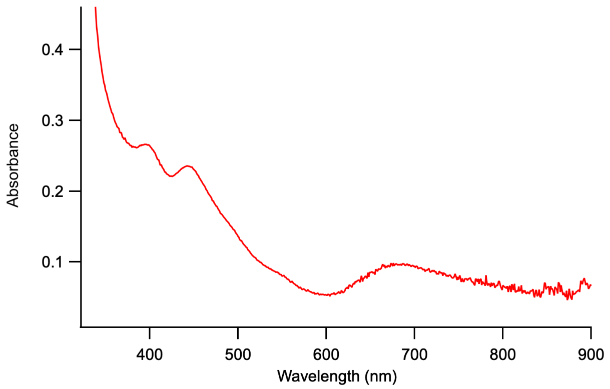

Figure S1: UV/vis spectrum of $Au_{25}(SC_6H_{13})_{18}^-$ without purification beyond that described here.

Ag-doped $Au_{25}(SC_6H_{13})_{18}^-$

A mixture of $Au_{25}(SC_6H_{13})_{18}^-$ and $Au_{24}Ag(SC_6H_{13})_{18}^-$ clusters was synthesized with a co-reduction of Ag and Au together in a mixture of THF. The synthetic procedure was exactly the same as for the Au_{25} clusters except the initial gold solution is replaced with a 200:1 $HAuCl_4$*$3H_2O$:$AgNO_3$ (0.51 mmol total metal concentration) solution in THF (20 mL).

More heavily Ag-doped clusters were synthesized with a co-reduction of Ag and Au together in a mixture of THF. The synthetic procedure was exactly the same as for the Au_{25} clusters except for an additional step 30 minutes after the addition of the thiol when $AgNO_3$ (0.036 mmol, 6.12 mg) was added in a 1:1 mixture of THF and water (5 mL).

Clusters with different ratios of dopants were produced by equilibration of mixtures of the solutions described above, according to the approach outlined by Pradeep and co-workers.[3] Mixtures were allowed to rest at room temperature for approximately 18 hours to ensure equilibration. Mass spectra after equilibration are presented in the following section.

Clusters protected by phenylethanethiol ligands were synthesized using the same procedure, simply replacing hexanethiol with phenylethanethiol.

Mass Spectrometry

Two different mass spectrometers were used for these studies. For routine characterization of raw synthetic products, a Bruker Compact quadrupole-time-of-flight instrument with high resolution was employed. As-synthesized products were dispersed in THF, directly infused via a syringe pump, and electrosprayed. Figures S2 and S3 were recorded as the average of one minute acquisitions. For electronic spectroscopy experiments, a home-built photo-fragmentation mass spectrometer (as described in the experimental details section) was employed. This instrument features lower resolution than the Bruker Compact and was not able to resolve the isotope patterns. However, it was able to clearly identify the dopant distributions as presented in Figure S4. Here, product mixtures were dispersed in acetone, directly infused via a syringe pump, and electrosprayed. Mass spectra as shown are the average of 1000 acquisitions.

Experimental Details

All electronic spectra were recorded on a home-built photofragmentation mass spectrometer.[4] Samples were prepared as described in the Mass Spectrometry section of this Supplementary Information. Ions were electrosprayed in a nitrogen atmosphere at room temperature and pressure and transferred into a vacuum system via a stainless steel capillary and skimmer. They were directed into a room temperature ion trap via an octopole ion guide and a

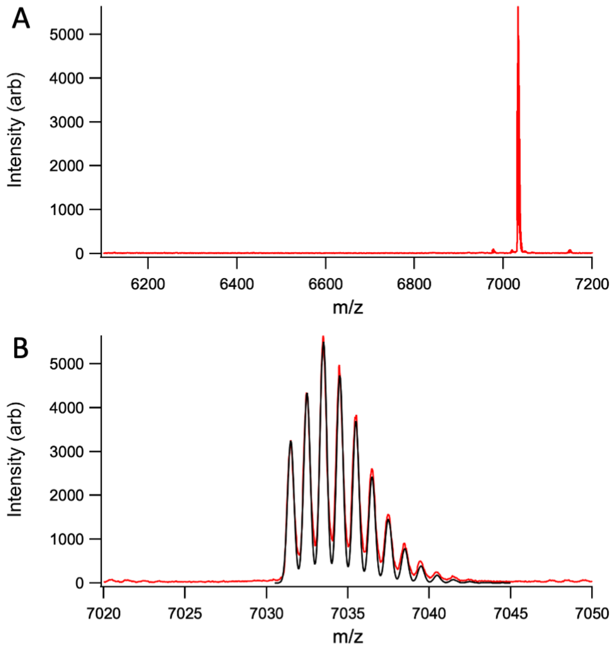

Figure S2: (A) Mass spectrum of the as-synthesized $Au_{25}(SC_6H_{13})_{18}^-$ nanoclusters. (B) Detailed view of the $Au_{25}(SC_6H_{13})_{18}^-$ peaks (red) compared to a simulation of the expected isotope pattern (black).

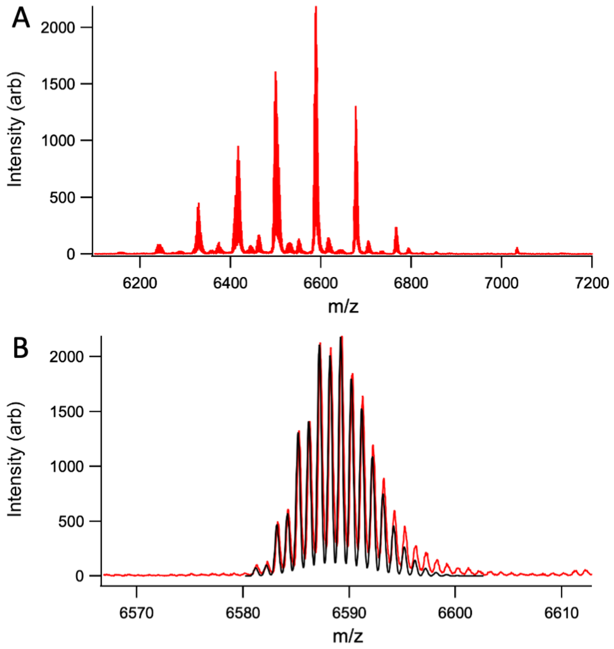

Figure S3: (A) Mass spectrum of the as-synthesized $Au_{25-n}Ag_n(SC_6H_{13})_{18}^-$ nanoclusters, showing cluster ranging from two to ten Ag dopants. Small peaks corresponding to doubly-charged dimer and triply-charged trimer species can be seen between the main dopant peaks. (B) Detailed view of the $Au_{20}Ag_5(SC_6H_{13})_{18}^-$ peaks (red) compared to a simulation of the expected isotope pattern (black).

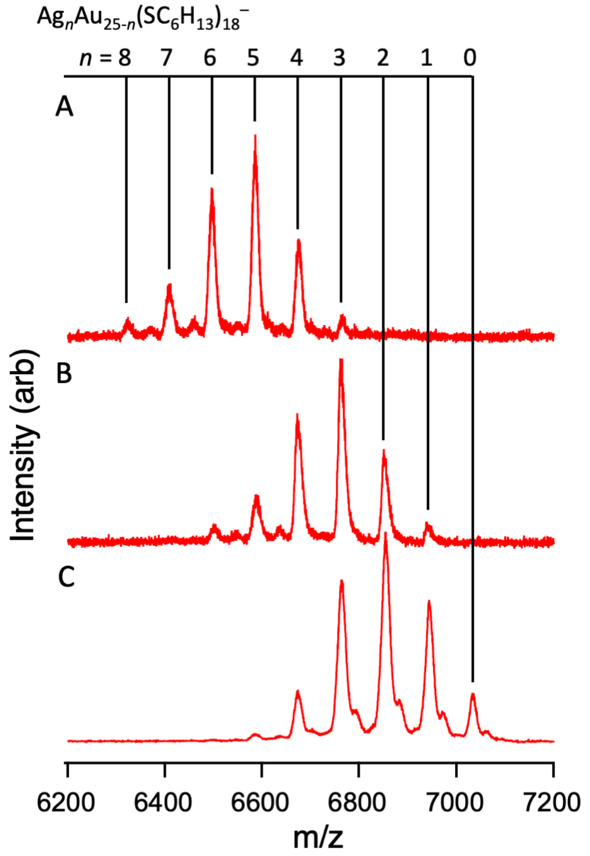

Figure S4: Mass spectra of the $Au_{25-n}Ag_n(SC_6H_{13})_{18}^-$ nanoclusters synthesized by mixing and equilibrating combinations of the syntheses shown in Figure S2 and Figure S3, for ratios of (A) 0:1, (B) 2:1, and (C) 1:1 (given as $Au_{25}(SR)_{18}^-$:$Au_{25-n}Ag_n(SC_6H_{13})_{18}^-$).

quadrupole mass filter set to pass all masses. After thermalization in the ion trap, the ions were transferred at a repetition rate of 10 Hz through another series of ion guides and a quadrupole bender before ultimately being stored in a cryogenic ion trap operating at 3.5-3.8 K. Here, the ions cooled in the presence of helium buffer gas to a temperature low enough to allow 20-30 He adduct atoms to physisorb on the clusters, with no clusters remaining without He adducts. After 99 ms of trapping time to allow cooling and adduct formation, a pulse from a tunable beta-Barium Borate-based optical parametric oscillator (LaserVision) pumped by a Nd:YAG laser (Continuum Surelite EX) was directed into the ion trap. When resonant with an optical transition, this leads to dissociation of all tags from the fraction of clusters absorbing a single photon. This results in the recovery of a population of clusters with no He adducts. The ratio of clusters without and with adducts is probed by extracting roughly 10% of the trapped ions into a time of flight mass spectrometry stage 10 μs after irradiation. This mass spectrum is digitized and the ratio of bare and adduct clusters is quantified by integrating over the respective peaks. For samples with mixtures of different numbers of Ag dopants, a single dopant peak (identified by mass) is monitored to achieve unambiguous absorption spectra of that specific composition. The laser pulse energy at each wavelength is recorded by a pyroelectric energy meter placed on the opposite side of the cryogenic ion trap. The presented spectra are then calculated by

$$S(\lambda) = \frac{F(\lambda)}{(F(\lambda) + P(\lambda))E(\lambda)} \tag{1}$$

where $F(\lambda)$ is the integrated signal of the clusters with no He adducts (fragments), $P(\lambda)$ is the integrated signal of the clusters with He adducts, and $E(\lambda)$ is the laser pulse energy. Spectra presented here are histogrammed with 1 nm bin widths and are the sum of 4-10 individual scans.

Computational Details

Optical properties for analogous $Au_{25-n}Ag_n(SH)^-$ clusters were evaluated by using the ADF code[5] incorporating both scalar and spin-orbit corrections via the ZORA Hamiltonian,[6] and the asymptotically correct potentials from the Leuween-Barends xc potential[7] (LB94) and TZ2P basis set, as employed in related coinage metal clusters.[8] Typical errors of TD-DFT calculation are about 0.15-0.30 eV in relation to experimental values.[9–11] Basis set of Triple-ζ Slater basis set, plus two polarization functions (STO-TZ2P) quality was employed. The frozen core approximation was applied to the [$1s^2$-$4p^6$] for Ag, [$1s^2$-$5p^6$] for Ag, [$1s^2$-$2p^6$] for S, leaving the remaining electrons to be treated variationally. Structures as $Au_{25-n}Ag_n(SH)^-$ were extracted from a previous study[12] which were optimized without any symmetry restrain via the analytical energy gradient method implemented by Versluis and Ziegler,[13] involving an energy convergence criterion of 10^{-4} Hartree, gradient convergence criteria of 10^{-4} Hartree/Å, and radial convergence criteria of 10^{-3} Å.

Estimation of the Optical HOMO-LUMO Gap

The optical HOMO-LUMO gaps for each spectrum were estimated by linear extrapolation of the most linear part of the low-energy side of the lowest-energy transition to determine the x-intercept. Linear fits were performed using Igor Pro 7.0 by Wavemetrics. Uncertainties presented are 1σ uncertainties determined by propagating the uncertainties of the slope and y-intercept for each fit, and represent the uncertainty of the extrapolation, not the uncertainty of the HOMO-LUMO gap itself. Figure S5 shows the results of the fits and Table S1 summarizes the extrapolated values.

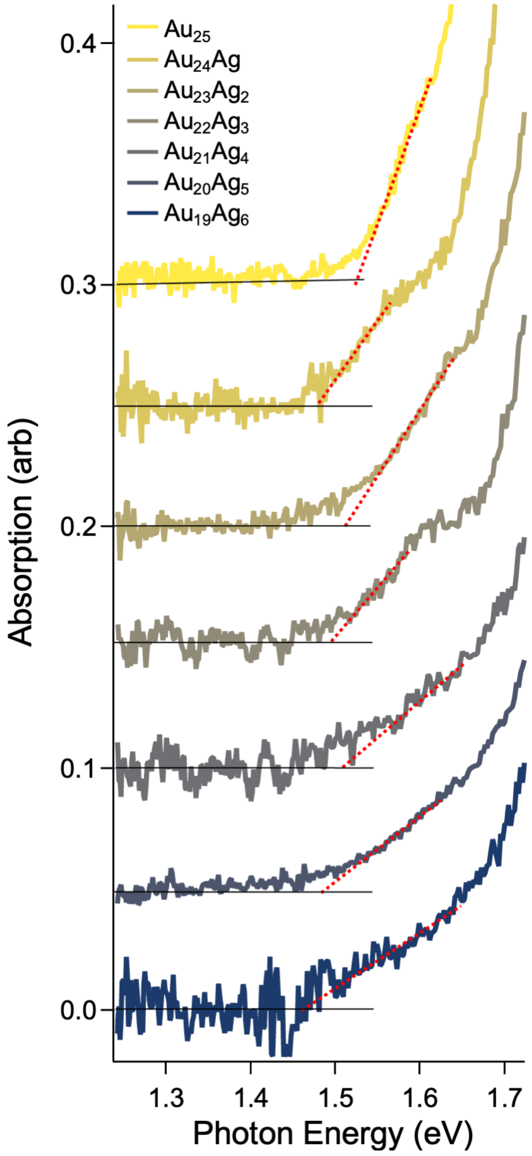

Figure S5: Extrapolation of the linear part of the low-energy edge of the absorption spectra to estimate the optical HOMO-LUMO gap.

Table S1: Extrapolated optical HOMO-LUMO gaps (in eV) for each cluster with 1σ uncertainties.

Cluster	Optical HOMO-LUMO Gap
Au_{25}	1.524 ± 0.001
$Au_{24}Ag_1$	1.479 ± 0.003
$Au_{23}Ag_2$	1.511 ± 0.002
$Au_{22}Ag_3$	1.489 ± 0.005
$Au_{21}Ag_4$	1.507 ± 0.007
$Au_{20}Ag_5$	1.489 ± 0.002
$Au_{19}Ag_6$	1.462 ± 0.005

Spectral Decomposition

Spectra were decomposed into six transitions by fitting to a model composed of the sum of a constant background and six Gaussian functions for the two low-energy peaks. For Ag-doped clusters, an additional Gaussian function was added to account for the first band of the putative Ag 5*d*-based transitions. Fitting was carried out using the Fityk program with the built-in Levenberg-Marquardt algorithm. The spectrum of $Au_{25}(SR)^-_{18}$ was fit first, and then the best-fitting values were used as initial guesses for the $Au_{24}Ag(SC_6H_{13})^-_{18}$ spectrum. This process was repeated for each more progressively doped cluster until $Au_{19}Ag_6(SC_6H_{13})^-_{18}$, at which point a satisfactory fit could not be found. The results of these fits are shown in Figures S6—S11, and the peak centers are summarized in Table S2.

We expect six total transitions, given that these transitions arise between three filled orbitals and two unfilled orbitals. For some spectra, a satisfactory fit could be found with only five peaks in the fit model. The sixth peak was included in the model to ensure that the spectra of each cluster were treated consistently. Given that Ag and Au contribute the same number of electrons to the superatomic orbitals, there is no spectroscopic justification to remove a transition from the model for some of the doped clusters. While including the sixth peak in the fitting model risks overfitting, we decided that this consistency was preferable, given that much of the analysis involves comparisons across dopant numbers.

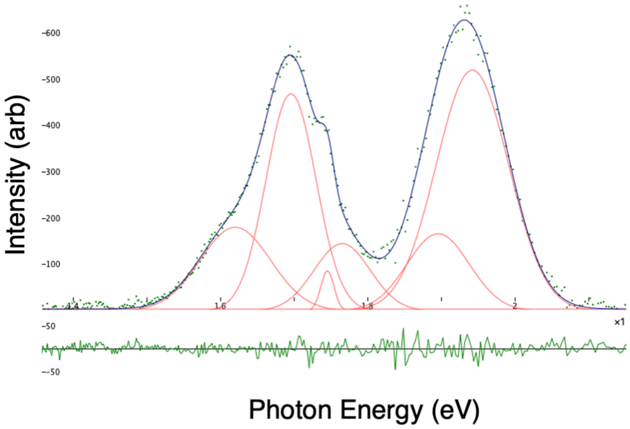

Figure S6: Best-fitting spectral decomposition of the $Au_{25}(SC_6H_{13})_{18}^-$ spectrum. All peaks are Gaussian functions, with a constant background.

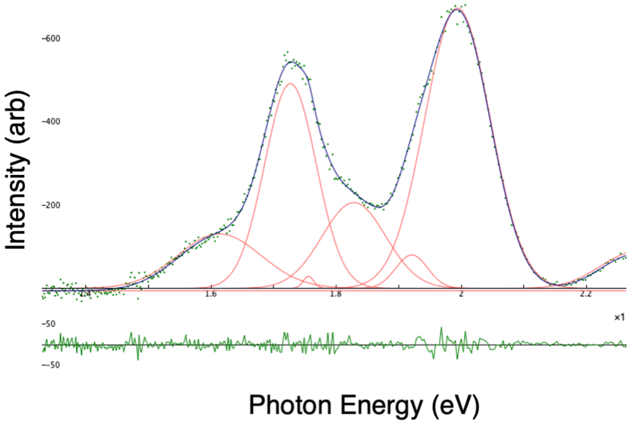

Figure S7: Best-fitting spectral decomposition of the $Au_{24}Ag(SC_6H_{13})_{18}^-$ spectrum. All peaks are Gaussian functions, with a constant background.

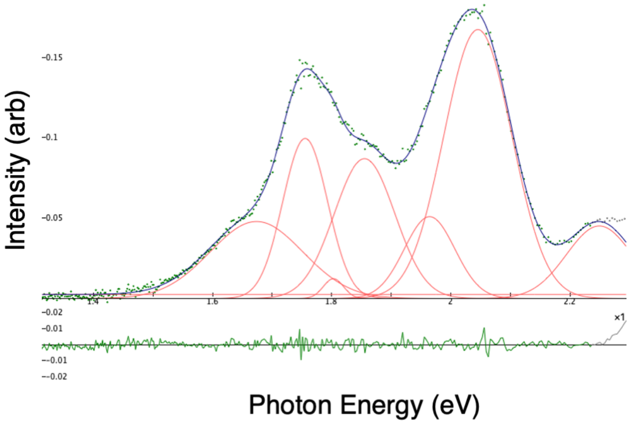

Figure S8: Best-fitting spectral decomposition of the $Au_{23}Ag_2(SC_6H_{13})_{18}^-$ spectrum. All peaks are Gaussian functions, with a constant background.

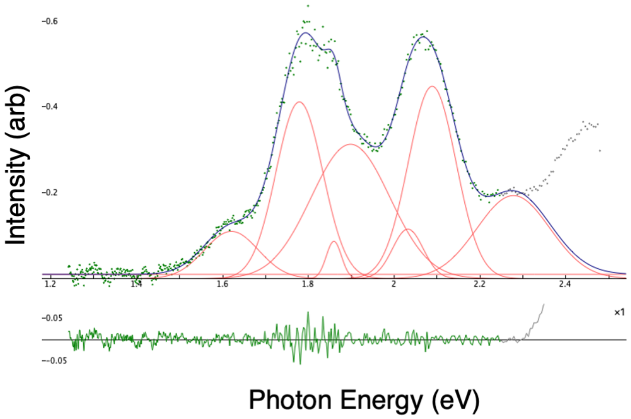

Figure S9: Best-fitting spectral decomposition of the $Au_{22}Ag_3(SC_6H_{13})_{18}^-$ spectrum. All peaks are Gaussian functions, with a constant background.

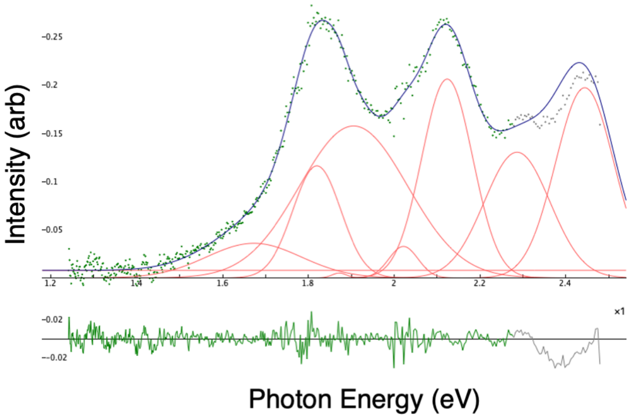

Figure S10: Best-fitting spectral decomposition of the $Au_{21}Ag_4(SC_6H_{13})_{18}^-$ spectrum. All peaks are Gaussian functions, with a constant background.

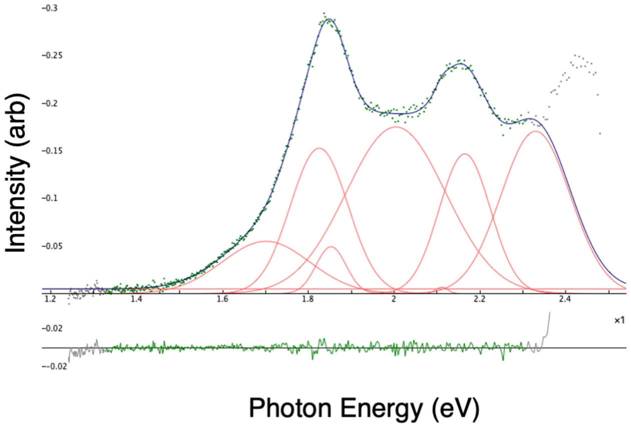

Figure S11: Best-fitting spectral decomposition of the $Au_{20}Ag_5(SC_6H_{13})_{18}^-$ spectrum. All peaks are Gaussian functions, with a constant background.

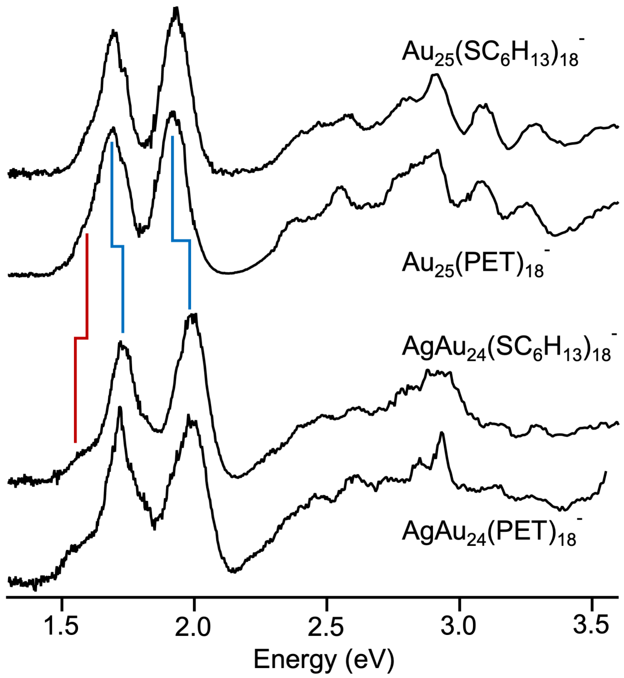

Figure S12: Comparison of the spectra of hexanethiol- and phenylethanethiol-protected clusters.

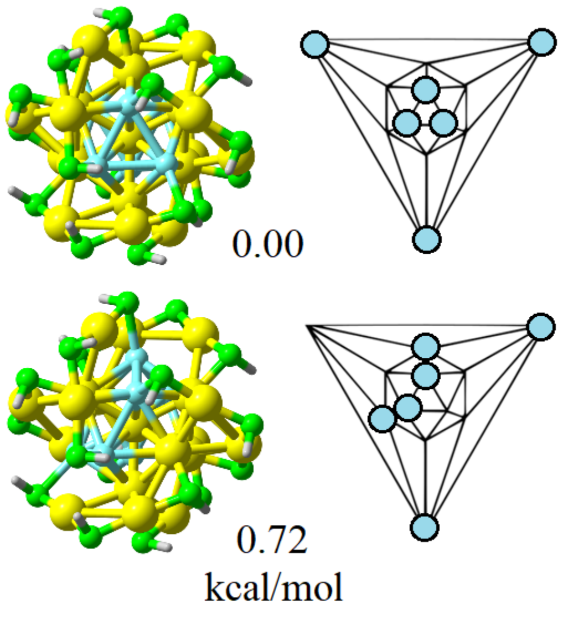

Figure S13: Structure for $Au_{19}Ag_6(SH)_{18}^-$ cluster, denoting the lowest two isomers (0.00 kcal/mol and 0.72 kcal/mol). In addition, Schlegel diagrams are provided to depict the silver atom array within the cluster, with light-blue spheres indicating the positions of the silver atoms. Color code. S: green, Au: yellow, Ag: light-blue, H: white.

Table S2: Best-fitting band centers (in eV) for the Gaussian components composing the fits shown in Figures S6—S11. Uncertainties given are 1σ uncertainties derived from the Hessian matrix at the best-fitting values.

Cluster	Peak 1	Peak 2	Peak 3	Peak 4	Peak 5	Peak 6
Au_{25}	1.619 ± 0.006	1.695 ± 0.006	1.745 ± 0.005	1.765 ± 0.025	1.895 ± 0.017	1.942 ± 0.013
$Au_{24}Ag_1$	1.613 ± 0.006	1.727 ± 0.001	1.756 ± 0.002	1.829 ± 0.003	1.921 ± 0.002	1.994 ± 0.001
$Au_{23}Ag_2$	1.674 ± 0.002	1.755 ± 0.002	1.803 ± 0.003	1.855 ± 0.004	1.964 ± 0.006	2.045 ± 0.005
$Au_{22}Ag_3$	1.620 ± 0.005	1.784 ± 0.002	1.860 ± 0.002	1.913 ± 0.011	2.028 ± 0.005	2.083 ± 0.006
$Au_{21}Ag_4$	1.673 ± 0.037	1.820 ± 0.008	1.872 ± 0.014	1.906 ± 0.052	2.022 ± 0.008	2.124 ± 0.005
$Au_{20}Ag_5$	1.701 ± 0.013	1.825 ± 0.024	1.853 ± 0.003	2.003 ± 0.018	2.111 ± 0.004	2.166 ± 0.006

References

(1) Zhu, M.; Aikens, C. M.; Hollander, F. J.; Schatz, G. C.; Jin, R. Correlating the Crystal Structure of A Thiol-Protected Au_{25} Cluster and Optical Properties. *J. Am. Chem. Soc.* **2008**, *130*, 5883–5885.

(2) Tofanelli, M. A.; Salorinne, K.; Ni, T. W.; Malola, S.; Newell, B.; Phillips, B.; Häkkinen, H.; Ackerson, C. J. Jahn–Teller effects in $Au_{25}(SR)_{18}$. *Chem. Sci.* **2016**, *7*, 1882–1890.

(3) Neumaier, M.; Baksi, A.; Weis, P.; Schneider, E. K.; Chakraborty, P.; Hahn, H.; Pradeep, T.; Kappes, M. M. Kinetics of Intercluster Reactions between Atomically Precise Noble Metal Clusters $[Ag_{25}(DMBT)_{18}]$ and $[Au_{25}(PET)_{18}]$ in Room Temperature Solutions. *J. Am. Chem. Soc.* **2021**, *143*, 6969–6980.

(4) Cirri, A.; Hernández, H. M.; Johnson, C. J. High Precision Electronic Spectroscopy of Ligand-Protected Gold Nanoclusters: Effects of Composition, Environment, and Ligand Chemistry. *J. Phys. Chem. A* **2020**, *124*, 1467–1479.

(5) Baerends, E. J. et al. The Amsterdam Modeling Suite. *J. Chem. Phys.* **2025**, *162*, 162501.

(6) van Lenthe, E.; Baerends, E. J.; Snijders, J. G. Relativistic total energy using regular approximations. *J. Chem. Phys.* **1994**, *101*, 9783–9792.

(7) van Leeuwen, R.; Baerends, E. J. Exchange-correlation potential with correct asymptotic behavior. *Phys. Rev. A* **1994**, *49*, 2421–2431.

(8) Bae, G.-T.; Aikens, C. M. Time-Dependent Density Functional Theory Studies of Optical Properties of Au Nanoparticles: Octahedra, Truncated Octahedra, and Icosahedra. *J. Phys. Chem. C* **2015**, *119*, 23127–23137.

(9) Weerawardene, K. L. D. M.; Aikens, C. M. Origin of Photoluminescence of $Ag_{25}(SR)_{18}^{-}$ Nanoparticles: Ligand and Doping Effect. *J. Phys. Chem. C* **2018**, *122*, 2440–2447.

(10) Ebina, M.; Iwasa, T.; Harabuchi, Y.; Taketsugu, T. Time-Dependent Density Functional Theory Study on Higher Low-Lying Excited States of $Au_{25}(SR)_{18}^{-}$. *J. Phys. Chem. C* **2018**, *122*, 4097–4104.

(11) Laurent, A. D.; Jacquemin, D. TD-DFT benchmarks: A review. *Int. J. Quantum Chem.* **2013**, *113*, 2019–2039.

(12) Knoppe, S.; Muñoz-Castro, A. Intermediate Silver Doping of $Au_{25}(SR)_{18}$: Variation of Electronic, Optical, and Chiroptical Properties along $Au_{25-x}Ag_x(SH)_{18}^{-}$ (x = 0–12) Stoichiometry from DFT Calculations. *Inorg. Chem.* **2023**, *62*, 7079–7086.

(13) Versluis, L.; Ziegler, T. The determination of molecular structures by density functional theory. The evaluation of analytical energy gradients by numerical integration. *J. Chem. Phys.* **1988**, *88*, 322–328.
